# Oxidation of arsenite to arsenate on birnessite in the presence of light

**DOI:** 10.1186/s12932-016-0037-5

**Published:** 2016-10-06

**Authors:** Samantha L. Shumlas, Soujanya Singireddy, Akila C. Thenuwara, Nuwan H. Attanayake, Richard J. Reeder, Daniel R. Strongin

**Affiliations:** 1 Department of Chemistry, Temple University, 1901 N. 13th St., Philadelphia, PA 19122 USA; 2 Department of Geosciences, Stony Brook University, Stony Brook, NY 11794 USA

**Keywords:** Arsenic remediation, Birnessite, Photochemistry, As(III) oxidation

## Abstract

The effect of simulated solar radiation on the oxidation of arsenite [As(III)] to arsenate [As(V)] on the layered manganese oxide, birnessite, was investigated. Experiments were conducted where birnessite suspensions, under both anoxic and oxic conditions, were irradiated with simulated solar radiation in the presence of As(III) at pH 5, 7, and 9. X-ray absorption spectroscopy (XAS) was used to determine the nature of the adsorbed product on the surface of the birnessite. The oxidation of As(III) in the presence of birnessite under simulated solar light irradiation occurred at a rate that was faster than in the absence of light at pH 5. At pH 7 and 9, As(V) production was significantly less than at pH 5 and the amount of As(V) production for a given reaction time was the same under dark and light conditions. The first order rate constant (k_obs_) for As(III) oxidation in the presence of light and in the dark at pH 5 were determined to be 0.07 and 0.04 h^−1^, respectively. The As(V) product was released into solution along with Mn(II), with the latter product resulting from the reduction of Mn(IV) and/or Mn(III) during the As(III) oxidation process. Post-reaction XAS analysis of As(III) exposed birnessite showed that arsenic was present on the surface as As(V). Experimental results also showed no evidence that reactive oxygen species played a role in the As(III) oxidation process.

**Electronic supplementary material:**

The online version of this article (doi:10.1186/s12932-016-0037-5) contains supplementary material, which is available to authorized users.

## Background

The metalloid arsenic is one of the most common contaminants in ground water, where it is derived mainly from oxidative weathering of rocks [1] and from industrial effluents [2, 3]. Arsenic in aquatic systems exists mainly in the 3+ and 5+ oxidation states, occurring primarily as arsenite (H_3_AsO_3_) and the oxyanion arsenate (AsO_4_^3−^), respectively. Although both forms pose health risks, the trivalent species is considered more toxic and more mobile than the pentavalent form [4]. The population of Bangladesh is most notably impacted by arsenic polluted drinking water with as many as 30 million of its citizens being regularly exposed [5]. Arsenic removal treatments are generally focused on reducing arsenic to acceptable levels; the World Health Organization has set a maximum allowed concentration of arsenic in drinking water at 10 μgL^−1^ (10 ppb) [6].

Oxidation of As(III) to As(V) and then sorption to a mineral surface is a strategy that aims to convert a toxic, mobile form of As to its less mobile form, with sorption allowing for subsequent removal of the pollutant. Photochemical processes that facilitate or enhance the oxidation of trivalent arsenic have received considerable interest in recent years. Semiconductors such as TiO_2_ are already in use in arsenic remediation schemes, although the activation of this particular material requires ultraviolet (UV) radiation for the oxidation of As(III) [7–11]. More recent work from our laboratory has shown that the oxidation of As(III) and the adsorption of the As(V) product in part can be carried out on the iron oxyhydroxides ferrihydrite and goethite in the presence of simulated solar light [12, 13]. Neither TiO_2_ nor the iron oxyhydroxides show significant activity toward As(III) oxidation in the absence of light.

Unlike TiO_2_ and iron oxyhydroxides, birnessite, the focus of the current study, can facilitate the oxidation of As(III) to As(V) in the absence of light [4, 14]. Birnessite, a layered manganese oxide containing both Mn^3+^ and Mn^4+^ within its structure, is composed of edge sharing MnO_6_ octahedra arranged into stacked sheets that are separated by cations [4, 14]. Depending on the position of the Mn^3+^ within the structure, triclinic or hexagonal birnessite can result. Manganese(III) that is present within the MnO_2_ sheet is characteristic of triclinic birnessite and the negative charge of the individual sheets is compensated by interlayer cations such as Na^+^ [15]. Hexagonal birnessite has Mn^3+^ in the interlayer above and below cation vacancies within the Mn^4+^ rich MnO_2_ sheets [16]. Interlayer regions can also contain Mn(II), other metal cations (M^+^/M^2+^) and H_2_O [17–19]. Both of these phases of birnessite have been found to facilitate the oxidation of As(III) to As(V) in the absence of light [4, 14, 20–27] as presented in the following composite reaction:

1 $$ {\text{MnO}}_{ 2} + {\text{ 2H}}^{ + } + {\text{ H}}_{ 3} {\text{As}}^{\text{III}} {\text{O}}_{ 3} ({\text{aq}}) \to {\text{Mn}}( {\text{II}}) \, ( {\text{aq}} ) + {\text{ H}}_{ 3} {\text{As}}^{\text{V}} {\text{O}}_{ 4} ( {\text{aq}}) + {\text{ H}}_{ 2} {\text{O}} $$

There have been a limited number of studies that have investigated the photochemical activity of birnessite, which is a small bandgap semiconductor [28–33]. We are unaware of any studies that have investigated the photochemistry of As(III) in the presence of birnessite. A brief survey of the literature shows that the band gap of birnessite has been measured in the range 1.8–2.7 eV [33–35]. Semiconductors have a filled valence band (VB) and empty conduction band (CB), and, when irradiated with light of energy greater than or equal to the band gap, an electron in the VB is excited to the CB, creating a hole in the VB. The resulting conduction band electron (${\text{e}}_{\text{cb}}^{ - }$) and valence band hole (${\text{h}}_{\text{vb}}^{ + }$) can be strong reducing and oxidizing agents, respectively.

In the present study we used batch geochemical techniques to compare the As(III) oxidation rate on triclinic Na-birnessite in the presence and absence of simulated solar radiation. The oxidation was investigated under different pH conditions (i.e., pH 5, 7, and 9) and in the presence and absence of oxygen. X-ray absorption spectroscopy (XAS) was used to determine the oxidation state of arsenic adsorbed on the birnessite. Solution phase experiments were also conducted to help shed light on whether reactive oxygen species (ROS) contributed to the photochemistry of As(III) in the presence of birnessite.

## Methods

### Synthesis and materials

All the chemicals used were obtained from commercial vendors and were used without any further purification. Nanopure deionized (DI) water (18.2 MΩcm^−1^) was used to prepare all the solutions and suspensions.

Na-birnessite used in this study was synthesized by using a modified version of the method utilized by Yang et al. [36]. Briefly, a 0.4 M Mn^2+^ solution was mixed with 8 M NaOH in 0.5 L of water (both solutions made in deionized/deoxygenated water), which was cooled to 0 °C and oxidized by bubbling oxygen through the solution for 5 h. The resulting suspension was aged at 90 °C for 48 h with constant stirring. The resulting product was vacuum filtered and washed multiple times with deionized water. The sample was then freeze-dried [18].

### Characterization methods

Synthetic birnessite was characterized using powder X-ray diffraction (XRD), Brunauer–Emmett–Teller (BET) specific surface area analysis, and transmission electron microscopy (TEM). XRD was carried out by using a Bruker AXS single crystal X-ray diffractometer with Mo Kα radiation and a graphite monochromator. Samples were prepared by placing the sample powder into glass capillaries. The XRD pattern of Na-birnessite (Additional file 1: Figure S1) was similar to the XRD pattern of samples synthesized and used in prior studies [18, 36–40]. Typical stoichiometries obtained from these synthetic methods are reported on in prior studies [38–40].

BET surface area was measured by single-point BET N_2_ adsorption using an ASAP 2020 surface area analyzer from Micromeritics. The birnessite sample was degassed at 150 °C for 2 h [41, 42]. The BET surface area of Na-birnessite used for all batch experiments was determined to be 18.90 m^2^g^−1^. The BET surface area of Na-birnessite used for XANES analysis was 23.10 m^2^g^−1^.

Samples for TEM analysis (JEOL JEM-1400, 120 kV) were prepared by suspending birnessite in deionized water and sonicating for 5 min. One drop of the suspension was deposited onto a 300 mesh carbon coated copper grid (Ted Pella, Redding, CA) and air dried. The morphology of the as-synthesized Na-birnessite particles (Additional file 1: Figure S2) was similar to the morphologies reported previously [18, 37]. We note here that samples investigated after irradiation looked similar to those before reaction (see Additional file 1: Figure S3).

### Batch experiments

All the dark and photochemical (light) experiments were conducted in a 200 mL water-jacketed beaker to prevent suspension heating during the 8 h experiments. Birnessite (30 mg) was suspended in 148.6 mL of DI water, sonicated for 20 min, and stirred for 30 min. The pH of the suspension was then adjusted to pH 5.0, 7.0, or 9.0. Next, 1.4 mL of a 50 mM As(III) (NaAsO_2_) solution was added to the suspension, making the total concentration of As(III) ~470 ± 10 µM. For the light experiments, the suspension of interest was immediately exposed to a 900 W high pressure Xe lamp, with a maximum light output at wavelength of ~600 nm [12] and a power density of 1450 Wm^−2^ (1.45 suns). The pH during the reaction was monitored using a Metrohm 718 STAT Titrino and was maintained through manual pH adjustment that was checked every 0.5 h. For both dark and light reactions, the suspensions were stirred throughout the experiment. Anoxic (no dissolved oxygen) batch studies were conducted by bubbling Ar gas (high purity, Airgas) though the solution of interest for 1 h prior to irradiation, and a constant Ar environment was maintained throughout these particular experiments. Oxic experiments were conducted in ambient air. After reaction, the birnessite suspensions from the pH 5 light and dark reactions were centrifuged and the bulk material was washed once with pH 5 DI water then air-dried for TEM, XRD, and XAS analysis.

Ion chromatography (IC, Dionex ICS1000) was used to determine the concentration of As(V) and Mn^2+^ in solution during the oxidation of As(III). All aliquots of the suspension were passed through 0.22 µm filters before being analyzed with IC. Only As(V) was detectable by IC. As(III) in solution was determined indirectly by adding H_2_O_2_ to the solution of interest (method adopted by Hansen et al. [43]), which oxidized As(III) in solution to As(V). IC of this solution yielded the total arsenic solution concentration (As(III) + As(V)). This value along with the As(V) concentration associated with the solution prior to oxidation was used to obtain the As(III) concentration prior to oxidation. Total adsorbed arsenic on birnessite was determined by subtracting the total arsenic present in solution (after H_2_O_2_ addition) from the known concentration of As(III) added initially.

### Detection of reactive oxygen species (ROS)

The possibility of hydroxyl radical (OH·) generation during the photochemical reactions was investigated with a fluorescence method using coumarin [44, 45]. The reaction of coumarin with hydroxyl radical yields 7-hydroxycoumarin, which gives a distinctive fluorescence peak at 460 nm upon excitation at 332 nm. Our experimental protocol involved the addition of coumarin to the reaction system to achieve a concentration of 1 mM prior to irradiation. Samples were then obtained at fixed times and they were analyzed in a fluorometer (Photon Technology International spectrometer) using 332 nm excitation radiation.

The addition of mannitol to certain reaction solutions was also carried out to supplement the coumarin studies. Prior studies have shown that mannitol is an effective scavenger of OH· and oxidative holes formed during the irradiation of semiconductor materials [46]. In these particular experiments, 20 mM mannitol was added to specific reaction mixtures of birnessite and As(III) and the effect of the mannitol addition on product formation was investigated under both light and dark conditions.

The possible presence of hydrogen peroxide in reaction solutions was investigated with an analytical technique that involved the use of 3′-(*p*-amino phenyl) fluorescein (APF) and Horseradish peroxidase (HRP) in a phosphate buffer (pH 7.4) [47]. The technique is based on the ability of HRP to catalyze the oxidation of APF by H_2_O_2_. The oxidized product exhibited fluorescence at 515 nm (excitation wavelength, 490 nm). The detection of H_2_O_2_ was carried out in an ex situ manner. Aliquots of the solution were periodically withdrawn from the system of interest and added to a buffered solution containing 10 µM APF and 0.2 µM HRP. An ex situ measurement was necessary for H_2_O_2_ detection, since in situ control experiments showed that exposure of the APF and HRP to light led to erroneous results, presumably due to the instability of HRP during irradiation.

Experiments were carried out to measure short-lived reactive oxygen species using electron paramagnetic resonance spectroscopy (EPR). Spectra were recorded at room temperature using a Bruker EMX-200R spectrometer at a microwave frequency of 3.65 GHz. Samples were prepared ex situ and then placed in a capillary tube inside of a quartz EPR tube for light irradiation. A fiber optic cable was used to irradiate the sample through the photolysis port on the EPR cavity. For in situ experiments a 10 mL aliquot was sampled out of the prepared batch experiment to which a spin trap was added (5,5-dimethyl-1-pyrroline-*N*-oxide, DMPO). Once in the EPR cavity, a dark spectrum was acquired and then the light was turned on. Successive scans were taken every 15 min for 1 h total. Experimental parameters included an attenuation of 5.0 dB, modulation frequency of 100 kHz, a modulation amplitude of 15 G, sweep time 20.97 s, receiver gain 2 × 10^4^. Data processing was done with Bruker software (WINEPR).

### X-ray absorption spectroscopy (XAS)

Arsenic K-edge X-ray absorption near-edge structure (XANES) spectroscopy was used to determine the average oxidation state of the arsenic sorbed on birnessite. In previous work from our laboratory [13], we showed that X-ray beam-induced oxidation of As(III) can be avoided by using quick-scanning X-ray absorption spectroscopy, as implemented at beamline X18B at the National Synchrotron Light Source, Brookhaven National Laboratory [13, 48, 49].

XANES spectra of As-reacted birnessite samples in the current study were collected over a duration of 30 s using the quick-scanning technique, with each scan requiring less than 1 s. As(III)-reacted samples were sealed between two layers of Kapton tape, and mounted at 45° relative to the incident X-ray beam. XANES spectra were measured in fluorescence mode, using a passivated implanted planar silicon detector. For each sample, the spectra within a sequence were compared to confirm the absence of any changes during scanning and then the first 3–5 spectra were averaged to achieve better signal/noise. A linear pre-edge background function was subtracted from the averaged spectrum, and normalization was performed at an energy value above the absorption edge (11,915 eV), as described by Bhandari et al. [50]. XANES spectra of As(III) and As(V) aqueous solutions were used as oxidation state references. The position of the absorption maximum for As(V) was found to be shifted ~3.5 eV higher than the position for the As(III) solution.

## Results and discussion

### Effect of light on the redox chemistry of As(III) on birnessite

#### Aqueous As(V) product

**Fig. 1:**
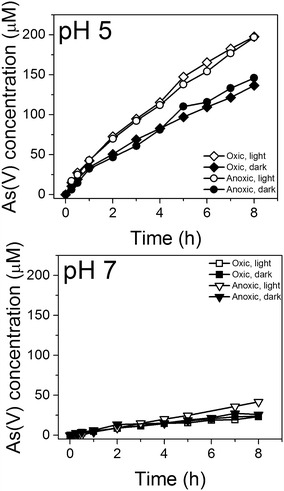
Oxidation of arsenite by Na-birnessite: the amount of arsenate released into the solution during the oxidation of As(III) (470 µM total) in the presence of Na-birnessite as a function of time in light and dark conditions at pH 5 and pH 7 under oxic and anoxic conditions

Figure 1 exhibits the solution phase concentration of As(V) as a function of time at pH 5 and 7 during the individual exposure of birnessite to 470 µM As(III) under dark and light conditions, and under anoxic and oxic solution conditions. We mention that control experiments (Additional file 1: Figure S4), where aqueous As(III) was exposed to light for 8 h (pH 5 and 7) in the absence of birnessite, showed no detectable As(V) product. This result taken together with data from Fig. 1 indicates that the photochemical oxidation of As(III) to As(V) in these experiments does not occur unless birnessite is present.

Analysis of the data presented in Fig. 1 shows that the amount of solution phase As(V) oxidation product was not a function of the dissolved oxygen level during the light-exposure for a particular solution pH. In the presence of dissolved oxygen at pH 5, 136 μM of As(V)_(aq)_ product formed after 8 h under dark conditions and 197 μM of As(V) was produced when the reacting system was exposed to light for 8 h. The As(V) concentrations after 8 h are similar for the oxic and anoxic experiments in the presence of light (197 and 196 μM, respectively) and also similar in the dark experiments (136 and 147 μM, respectively). These results show that light irradiation has a greater influence on the oxidation As(III) and oxygen does not appear to play a major role in the oxidation mechanism.

The total arsenic in solution did not change significantly over the course of the experiment, indicating that As did not sorb to the surface at an experimentally observable amount. Based on the As(III) loss from solution, which was determined by subtracting the As(V) produced over time from the amount of As initially added (470 μM), we calculate the first order rate constant (k_obs_) for As(III) oxidation to be 0.07 and 0.04 h^−1^, respectively, under light and dark conditions (dissolved oxygen was present). We point out that the measured k_obs_ in our dark experiment at pH 5 (0.04 h^−1^) is higher than the k_obs_ (0.02 h^−1^) reported previously by Tournassat et al. [27], using a crystalline hexagonal birnessite at pH 5. One likely reason for this discrepancy is the nature of the birnessite samples, since significant variations can occur depending of the crystallinity, average oxidation state, vacancy concentration and morphology of the birnessite used. Also, the As:Mn ratio used in our experiment (0.22) was lower than in the prior study (0.44).

Upon raising the pH of the reaction medium to 7 and 9 (Additional file 1: Figure S5) the amount of As(V) produced decreased relative to the amount of As(V) produced at pH 5 at the same birnessite loading. The amount of As(V) produced at pH 7 in the presence of light (23 μM) is approximately 0.1 times less than the amount experimentally observed at pH 5 in the presence of light (197 µM). At pH 9, the amount of As(V) produced over time is below the detection limit of the chromatographic method used to analyze for As(V). The final As(V) concentration after 8 h is presented in Table 1. The lower As(III) oxidation rate at higher pH conditions has been observed by others, and it is often attributed to the passivation of the birnessite surface by either manganese(II) or (III) [23, 25–27, 51, 52]. Also we note that the effect of light seems to be absent at these higher pH conditions, which could be due to this surface passivation.

**Table 1:** As(V) in solution after 8 h at pH 5, 7, and 9

	pH 5	pH 5	pH 7	pH 7	pH 9	pH 9
	Light	Dark	Light	Dark	Light	Dark
Oxic	197	136	23	23	–	6
Anoxic	196	145	43	25	13	–

Concentrations are given in µM

The general shape of the As(V) versus time plots (Fig. 1) associated with the photochemical system is qualitatively similar to the dark reaction. In particular, both reaction systems show an As(V) formation rate that decreases over time. Prior studies of the dark reaction system generally attributed this decrease to the passivation of the birnessite surface by Mn(III), derived from the reduction of two Mn(IV) species to Mn(III) by As(III) with the concomitant formation of As(V) [4, 23, 53]:

2 $$ 2 {\text{MnO}}_{ 2} + {\text{ H}}_{ 3} {\text{AsO}}_{ 3} + {\text{ H}}_{ 2} {\text{O}} \to 2 {\text{MnOOH }} + {\text{ H}}_{ 2} {\text{AsO}}_{ 4}^{ - } + {\text{ H}}^{ + } $$

More recent studies, however, show strong evidence that at early reaction times the primary reaction product is Mn(II), resulting from the 2-electron reduction of Mn(IV) by As(III) [51]:

3 $$ {\text{MnO}}_{ 2} + {\text{ H}}_{ 3} {\text{AsO}}_{ 3} + {\text{ H}}^{ + } \to {\text{Mn}}\left( {\text{II}} \right) + {\text{ H}}_{ 2} {\text{AsO}}_{ 4}^{ - } + {\text{ H}}_{ 2} {\text{O}} $$

It has been proposed that the primary buildup of Mn(III), which leads to passivation of the surface, results from the comproportionation reaction between Mn(II) product and surface Mn(IV) to produce Mn(III) [54, 55]. This increase in Mn(III) is evidenced by the transformation of triclinic birnessite to hexagonal birnessite (Additional file 1: Figure S6) [56], which is caused by migration of Mn(III) within the sheet to the interlayer region [39]. The oxidation of As(III) by surface Mn(III) is considered to be slow, consistent with the decreasing rate of As(V) formation as the reaction time increases [23, 51].

**Fig. 2:**
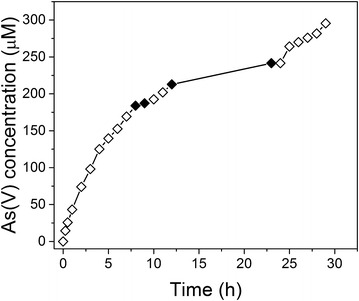
On/off cycling of light effects on As(III) oxidation: As(V) production resulting from a As(III)/birnessite suspension at pH 5 under oxic conditions. The open symbols indicate times that the suspension was exposed to light and the closed symbols indicate times where the light was turned off. Even at relatively long reaction times light enhances the formation rate of As(V)

We conducted an additional experiment to show that light continues to enhance the rate of As(V) production at reaction times where passivation of the birnessite surface has occurred (Fig. 2). In this experiment, conducted at pH 5, an As(III)/birnessite suspension was first exposed to light, and was followed by two off/on light cycles (indicated in the figure). The experimental strategy here was to best compare the rate of As(V) formation under light and dark conditions at extended reaction times. Analysis of the slopes associated with the plot during the light-off and light-on cycles shows that the rate of As(V) formation on the passivated birnessite is enhanced by a factor of 2.3 in the presence of light (relative to the dark data). In particular, the rate of As(V) production for the 8–10 h period (light off cycle) was 4.34 µM h^−1^ (12.52 µM h^−1^m^−2^), and this rate increased to 10.07 µM h^−1^ (29.07 µM h^−1^m^−2^) for the 10–12 h period when the sample was exposed to light. In the second light off/on cycle, the As(V) formation rate was 2.39 µM h^−1^ (6.91 µM h^−1^m^−2^) for the 12–24 h light-off period and increased to 10.76 µM h^−1^ (31.07 µM h^−1^m^−2^) for the 24–29 h light-on interval. While the rate of As(III) oxidation decreases over the course of time in the dark due to changes to the birnessite surface structure, exposure of the system to light still leads to an enhancement of As(III) oxidation rate relative to dark conditions. We come back to a brief discussion of this experimental observation later.

#### Adsorbed product on birnessite under dark and light conditions

During the course of the experiment, there was no major change to the total arsenic present in solution, therefore, a quantifiable amount of adsorbed product could not be determined in the aqueous batch reactions. This lack of quantifiable adsorbed arsenic could be due to the Na-birnessite sample possessing a small concentration of cation vacancies, which is the favored site for metal ions to sorb [39, 57]. This observation agrees with a recent study that investigated As(III) oxidation on different manganese oxides in which the experimental observation that crystalline triclinic birnessite did not adsorb observable amounts of As was also made [14]. Results from a different batch of Na-birnessite synthesized in our laboratory exhibited a higher surface area (HSA birnessite, 23.10 m^2^g^−1^) and led to an increased amount of As(V) adsorption during the As(III) oxidation reaction. We note that while the two different Na-birnessite samples showed differences in As(V) adsorption, both samples showed an enhancement in As(III) oxidation rate when exposed to light (data for HSA birnessite, shown in Additional file 1: Figure S7).

**Fig. 3:**
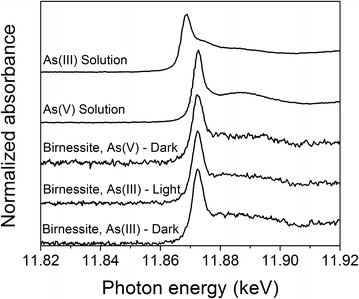
Characterization of surface species after reaction: arsenic K-edge XANES spectra of HSA Na-birnessite exposed to As(III) for 8 h under dark and light conditions. Also shown is the arsenic K-edge XANES spectrum of HSA Na-birnessite exposed to As(V) under dark conditions for 8 h, and spectra for As(III) and As(V) solutions. (All the plots were offset for clarity)

Arsenic K-edge XANES spectra presented in Fig. 3 indicate that the primary adsorbed product on the HSA birnessite after exposure to As(III) for 8 h is As(V) whether the reaction occurs in the absence or presence of light. In particular, the energy of the main peak in the XANES for all birnessite samples that were exposed to As(III) under all experimental conditions is identical to the value for the reference As(V) solution sample. This result is consistent with prior studies that have shown that As(III) oxidizes to As(V) on birnessite under dark conditions [4, 26, 27]. Our data show that under light conditions, As(V) is also the primary adsorbed product when birnessite is exposed to As(III).

#### Mn(II) product

**Fig. 4:**
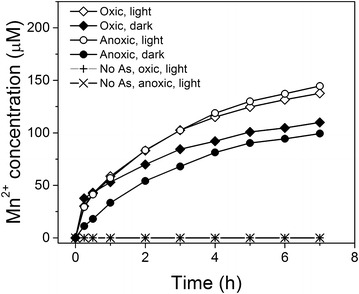
Manganese (II) release into solution: the concentration of dissolved Mn^2+^ released during the oxidation of 470 µM of As(III) by Na-birnessite at pH 5 in the presence of light and dark under oxic and anoxic conditions. Control experiments are also included for Na-birnessite with no As(III) at pH 5 in the presence of light under oxic and anoxic conditions

Figure 4 exhibits the concentration of Mn(II) product in solution as a function of time during the As(III) oxidation reaction (pH of 5) in the absence and presence of light for oxic and anoxic conditions. The amount of Mn(II) partitioning into solution is higher in the presence of light than in the dark. After 7 h of reaction (under oxic conditions), the concentration of dissolved Mn(II) is 137 and 109 μM, in the light and dark experiments, respectively. Using these Mn(II) data along with the concentration of oxidized As(III) (i.e., As(V)_aq_ + As(V)_ads_) we calculate Mn(II)_(aq)_:As(V) ratios of 0.75:1 and 0.90:1 for the light and dark experiments, respectively. The ratio associated with the dark reaction is close to the 1:1 stoichiometry associated with Eq. 1, whereas the ratio for the irradiated sample is lower. The difference could be due to the oxidation of Mn^2+^ to insoluble Mn(III) or Mn(IV) species in the presence of dissolved oxygen and light or the formation of ternary complexes containing Mn^2+^ and As(V) in the presence of light [25]. We note that control experiments, where birnessite suspensions in the absence of As(III) at pH 5.0 were irradiated, showed no detectable aqueous Mn(II) product. Also Mn(II) release at pH 7 and 9 was not experimentally observed in our studies, presumably due to the tendency of Mn(II) to stay surface bound at these higher pH conditions [26].

### Mechanistic aspects of As(III)/birnessite photochemistry

Relative to the reaction of As(III)/birnessite in the dark, the photochemistry of As(III)/birnessite system brings forward additional reaction pathways via the production of reducing ${\text{e}}_{\text{cb}}^{ - }$ and oxidizing ${\text{h}}_{\text{vb}}^{ + }$. With regard to the reducing conduction band electrons, prior research has shown that the irradiation of MnO_2_ in the aqueous environment results in the release of Mn(II), redox chemistry that can be expressed by the following composite reaction [34, 58]:

4 $$ {\text{MnO}}_{ 2} + {\text{ 4H}}^{ + } + {\text{ 2e}}_{\text{cb}}^{ - } \to {\text{ Mn}}\left( {\text{II}} \right)  + {\text{ 2H}}_{ 2} {\text{O}} $$

Furthermore, studies have generally studied the production of Mn(II) photoproduct during the irradiation of manganese oxides in the presence of electron donors (e.g., organic species) that are oxidized by the photogenerated ${\text{h}}_{\text{vb}}^{ + }$. Interestingly, recent studies have also shown that the photogeneration of Mn(II) during the irradiation of birnessite can occur in an ice matrix in the absence of an electron donor (other than potentially water) [58], albeit at a much lower rate than if an electron donor was present. Recent studies investigated the photochemistry of birnessite with time-resolved XAS during the irradiation of the material with 400 nm light in water [59]. This particular study showed that the photogenerated electrons resulted in the reduction of Mn(IV) to Mn(III) that migrated into the interlayer region of the layered birnessite. It was speculated in this study that the oxidative hole could lead to the generation of reactive oxygen species such as hydroxyl radical, but their potential reaction to form H_2_O_2_ would only lead to the oxidation of Mn(III) back to Mn(IV) [59].

In the present study we suspect that the oxidative hole formed in the valence band of birnessite during irradiation was directly responsible for the oxidation of As(III). In short, our results do not give support to a scenario where ROS formed during the irradiation of birnessite in the presence or absence of As(III). Experiments were carried out that used both fluorescent probes and EPR (coupled with trapping agents) to investigate the production of ROS. To investigate the generation of hydroxyl radical, a fluorescence method using coumarin was employed [44, 45]. The reaction of coumarin with hydroxyl radical forms a fluorescent adduct with a unique emission. Data obtained using this method in situ (Additional file 1: Figure S8) did not show evidence for the presence of the adduct. We also used the APF-HRP test (see experimental) to detect hydrogen peroxide in solution, but also found no evidence for this species (Additional file 1: Figure S9). We point out, however, that if H_2_O_2_ was produced it might be expected to rapidly decompose in the presence of birnessite [60–62].

In addition to fluorescent-based probes, EPR experiments were carried out to further investigate the possibility of ROS generation. These particular experiments using DMPO as a spin-trapping agent for hydroxyl radical did not yield any support for the generation of this particular radical. Whether the experiment was carried out on an aqueous suspension of birnessite or suspension of birnessite in the presence of As(III), the resulting spectra could be associated with the characteristic EPR spectrum for Mn(II) (Additional file 1: Figures S10, S11). Consistent with our batch studies the magnitude of the Mn(II) spectral weight from the EPR experiment was greater when As(III) was present, compared to the irradiation of birnessite in As(III)-free water (Additional file 1: Figure S11). We attribute the increased Mn(II) signal to the presence of the electron donor (i.e., As(III)) that can be oxidized by the valence hole.

**Fig. 5:**
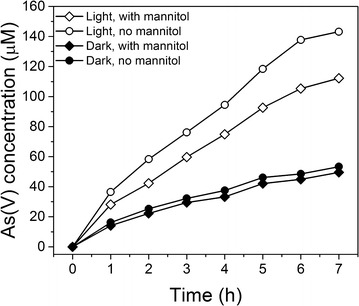
Effect of mannitol on the oxidation of arsenite by Na-birnessite: concentration of aqueous As(V) released into the solution during the oxidation of As(III) in the presence of Na-birnessite at pH 5 (oxic) under light and dark conditions with and without mannitol

To better determine whether As(III) oxidation occurred in part due to the presence of valence band holes in the presence of light, we carried out experiments that utilized mannitol. Prior studies have shown that mannitol is an efficient scavenger of OH· and oxidative holes [46]. Figure 5 shows data from an experiment where 20 mM mannitol was added to particular reaction mixtures. It is mentioned that both the coumarin-based analytical technique and EPR studies strongly suggest that solution OH· is not an important intermediate species when birnessite was irradiated in the absence or presence of As(III). Hence, we attribute mannitol-induced changes in the rate of production of As(V) in the presence of As(III), birnessite, and light to the scavenging of oxidative holes (or OH· formed through the oxidation of surface hydroxyl groups). It should be noted that the data show that the addition of mannitol, even at the relatively high concentration of 20 mM, does not affect As(V) product formation in the dark. We infer from this result that mannitol does not block birnessite sites that can oxidize As(III) to As(V) in the absence of light. In the photo-reaction data, the presence of mannitol results in an As(V) concentration that is approximately 30 % lower relative to the mannitol-free system after 7 h. We infer from this result that mannitol is scavenging photogenerated holes (that oxidize mannitol) that would otherwise oxidize As(III).

The mechanism by which As(III) is oxidized to As(V) photochemically is not entirely clear. One possible reaction sequence would be the oxidation of As(III) via two one-electron transfer steps as described by the following reactions [63, 64]:

5 $$ {\text{h}}_{\text{vb}}^{ + } + {\text{ As}}( {\text{III}}) \to {\text{As}}( {\text{IV}}) $$

6 $$ {\text{h}}_{\text{vb}}^{ + } + {\text{ As}}( {\text{IV}}) \to {\text{As}}( {\text{V}}) $$

If such a mechanism were operative it might be expected to exhibit rapid kinetics, since prior studies have shown that the reaction of As(IV) with dissolved O_2_ is a rapid reaction that leads to As(V) [65]. We, however, do not observe a dependence of the photochemical As(V) production rate on the presence of dissolved oxygen. It is conceivable that a species such as H_2_O_2_ could play a role in the oxidation of As(III) to As(V). If a short lived H_2_O_2_ ROS does play a role, it would be expected to form from chemistry initiated by the valence band hole and the oxidation of surface hydroxide [59]. Formation pathways via the reduction of dissolved oxygen do not appear to be a possibility, because of the aforementioned insensitivity of photochemical As(III) oxidation to dissolved oxygen. Whether this pathway is inhibited due to the reduction potential of oxygen lying higher than the conduction band minimum of birnessite, or to the efficiency of Mn(IV) [or Mn(III)] as an electron acceptor (to form Mn(III) and Mn(II), respectively) cannot be discerned from our results.

It is interesting that the photochemical oxidation of As(III) still occurs with a significant rate even when As(III) oxidation in the dark has decreased significantly after 20 h of reaction (Fig. 2). Most prior studies have shown for the dark reaction between As(III) and birnessite that the surface becomes covered with Mn(III) [23, 25–27, 52, 54]. It is conceivable that the irradiation of such a surface might not directly oxidize As(III) through valence band hole formation, but instead the oxidation of a fraction of Mn(III) species back to Mn(IV) by the hole could occur. As(III) oxidation on such newly created Mn(IV) sites would be expected to occur with faster kinetics than on Mn(III) sites [51]. The exact role of these species in the photochemistry of birnessite has significant implications in environmental manganese cycling. Understanding the mechanistic aspects of this chemistry warrants future study.

## Conclusions

We have shown that the oxidation of As(III) by Na-birnessite can be enhanced via irradiation with simulated solar light (at a solution pH of 5). The simulated light intensity used in this study is comparable to the light intensity experienced by the Earth’s surface environment (i.e., 1.45 suns). The photochemical pathways presented in this contribution are informed by the many prior studies that have investigated the adsorption and oxidation of As(III) on birnessite [4, 14, 23–27] under dark conditions. We propose that the creation of the hole-electron pair during the irradiation of the small band-gap semiconductor drives the oxidation chemistry; the valence band hole leads to the oxidation of As(III) and the conduction band electron leads to the reduction of Mn(IV)/Mn(III) and the formation of Mn(II) product. The results of this study have environmental implications, most notably that the birnessite-facilitated oxidation of mobile As(III) to the less mobile As(V) at appropriate pH conditions (pH 5 in our study) can be facilitated by photochemical means. Such a process could potentially be useful for a more efficient remediation of arsenic in environments where birnessite can absorb photons having a requisite energy. Future work that investigates the photochemistry of hexagonal birnessite would give insights on the effect that birnessite phase and crystallinity has on this chemistry as well.

## Additional file

10.1186/s12932-016-0037-5 Supporting information showing the XRD of birnessite, TEM images of birnessite, control experiments of As(III) irradiated in the absence of birnessite, pH 9 batch reaction data, post-reaction TEM images of birnessite, data relevant to the detection of OH and H_2_O_2_, and EPR data for As(III)/birnessite.
