# Supplementary material for: Oxidation of arsenite to arsenate on birnessite in the presence of light
Source: Geochem Trans. 2016 Oct 6;17:5. doi: 10.1186/s12932-016-0037-5 (PMC5053027; doi:10.1186/s12932-016-0037-5)
Supplement: Supplementary file 1 — 10.1186/s12932-016-0037-5 Supporting information showing the XRD of birnessite, TEM images of birnessite, control experiments of As(III) irradiated in the absence of birnessite, pH 9 batch reaction data, post-reaction TEM images of birnessite, data relevant to the detection of OH and H2O2, and EPR data for As(III)/birnessite. [file 12932_2016_37_MOESM1_ESM.pdf]

Supporting Information

Oxidation of arsenite to arsenate on birnessite in the presence of light

Samantha L. Shumlas[1], Soujanya Singireddy[1], Akila C. Thenuwara[1], Nuwan H. Attanayake[1], Richard J. Reeder[2], Daniel R. Strongin[1*]

[1]Department of Chemistry, Temple University, 1901 N. 13th St., Philadelphia, Pennsylvania 19122, United States

[2]Department of Geosciences, Stony Brook University, Stony Brook, NY 11794, United States

Email addresses:

Samantha L. Shumlas: sshumlas@temple.edu

Soujanya Singireddy: soujanyasingireddy@gmail.com

Akila Chathuranga Thenuwara: akila.thenuwara@temple.edu

Nuwan H. Attanayake: nuwan@temple.edu

Richard J. Reeder: rjreeder@stonybrook.edu

Daniel R. Strongin*: dstrongi@temple.edu

Supporting Information:

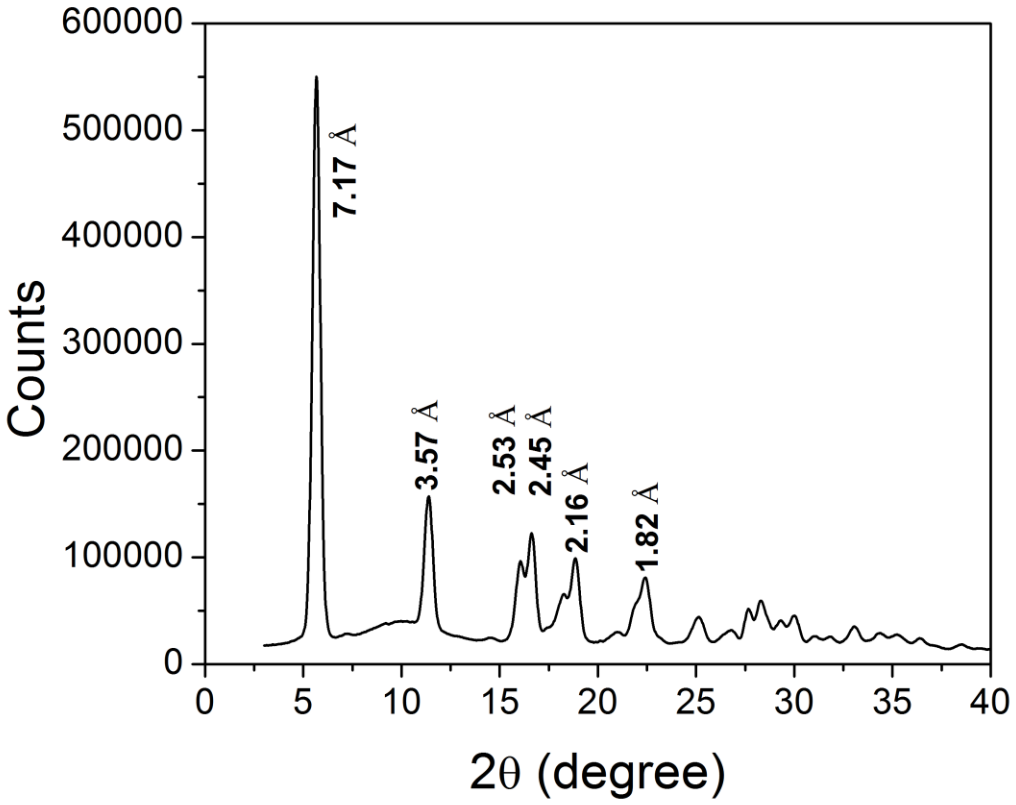

Figure S1. XRD pattern of Na-birnessite. (Mo Kα radiation)

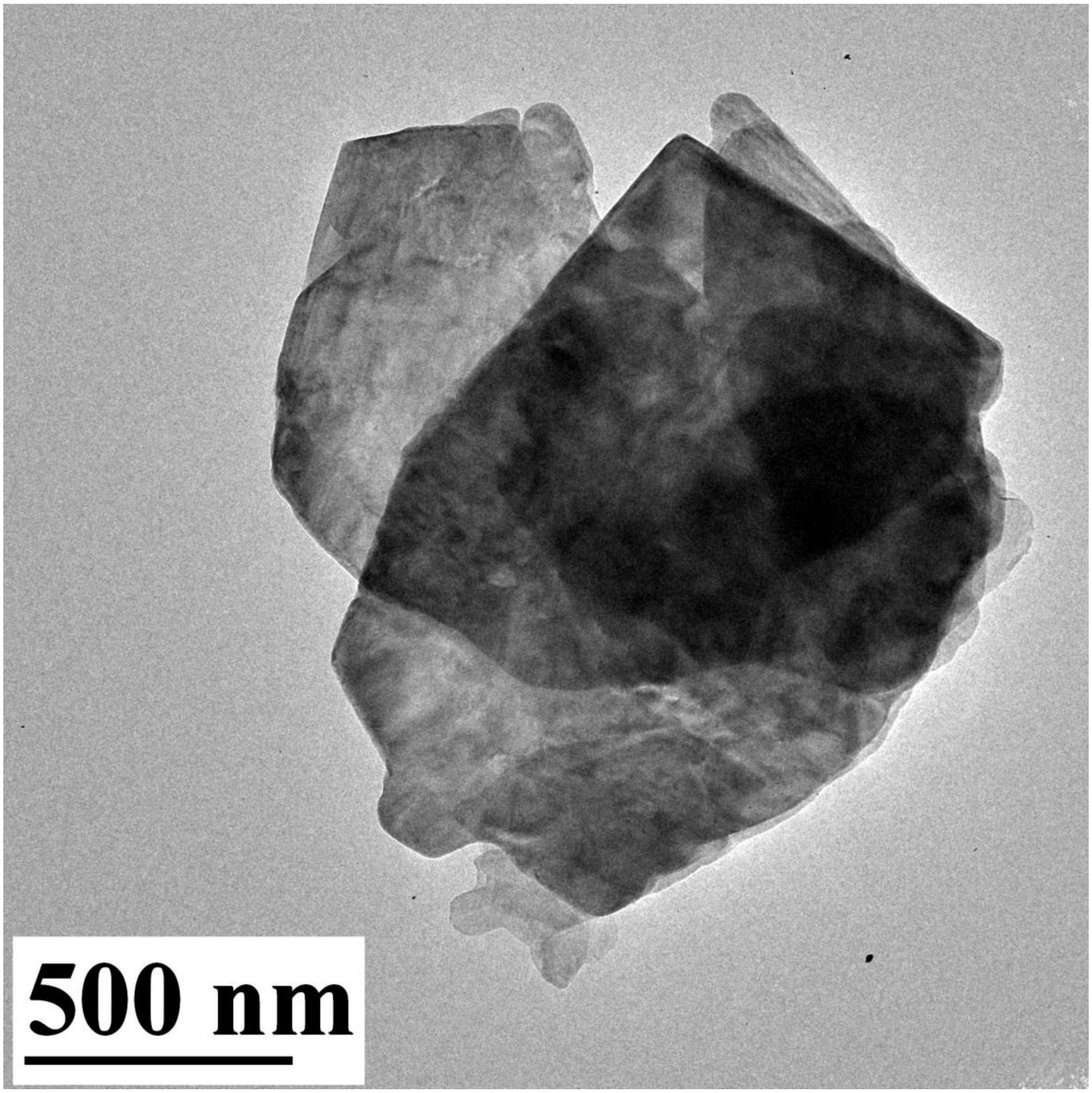

Figure S2. TEM image of birnessite starting material.

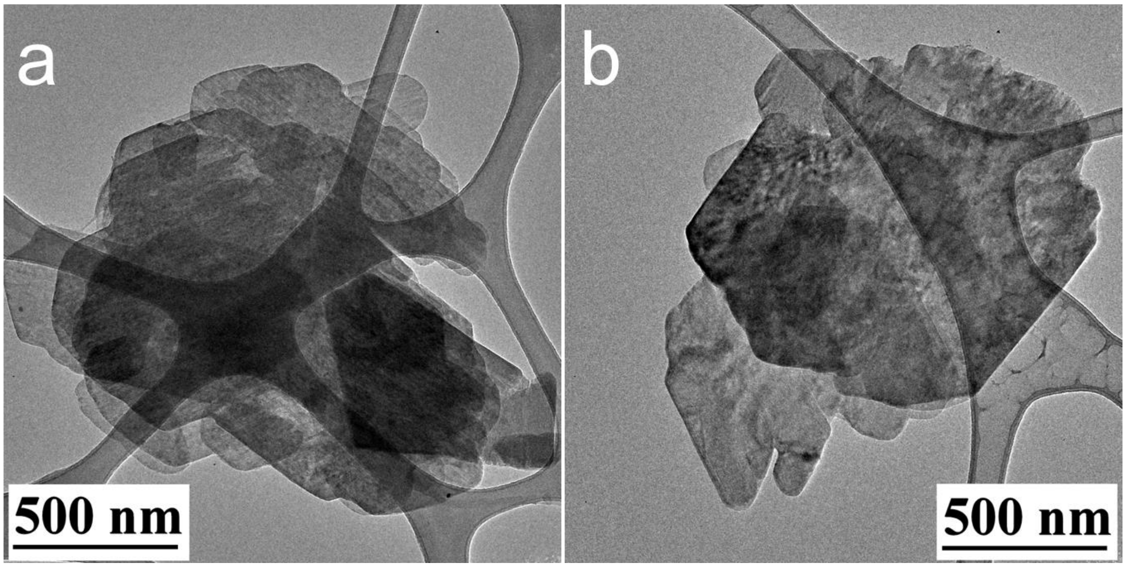

Figure S3: TEM images of the Na-birnessite particles after reaction with arsenite at pH 5 for (a) 8 h in dark, (b) 8 h in light.

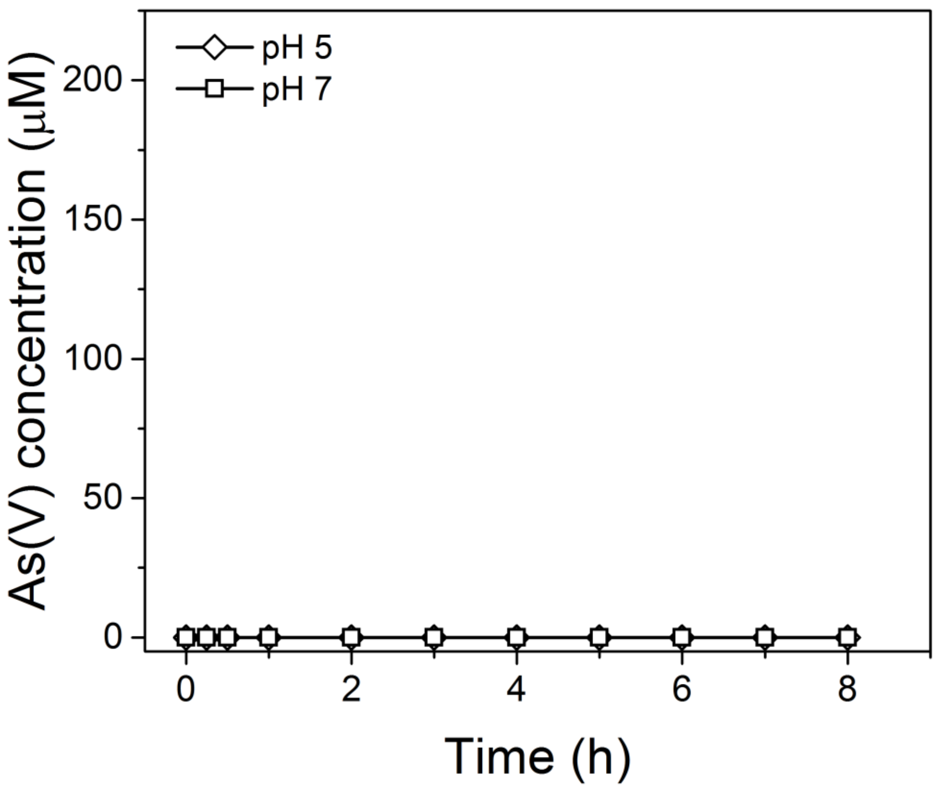

Figure S4. Concentration of aqueous As(V) formed in solution during the irradiation of aqueous As(III) at pH 5 and 7, in the absence of birnessite.

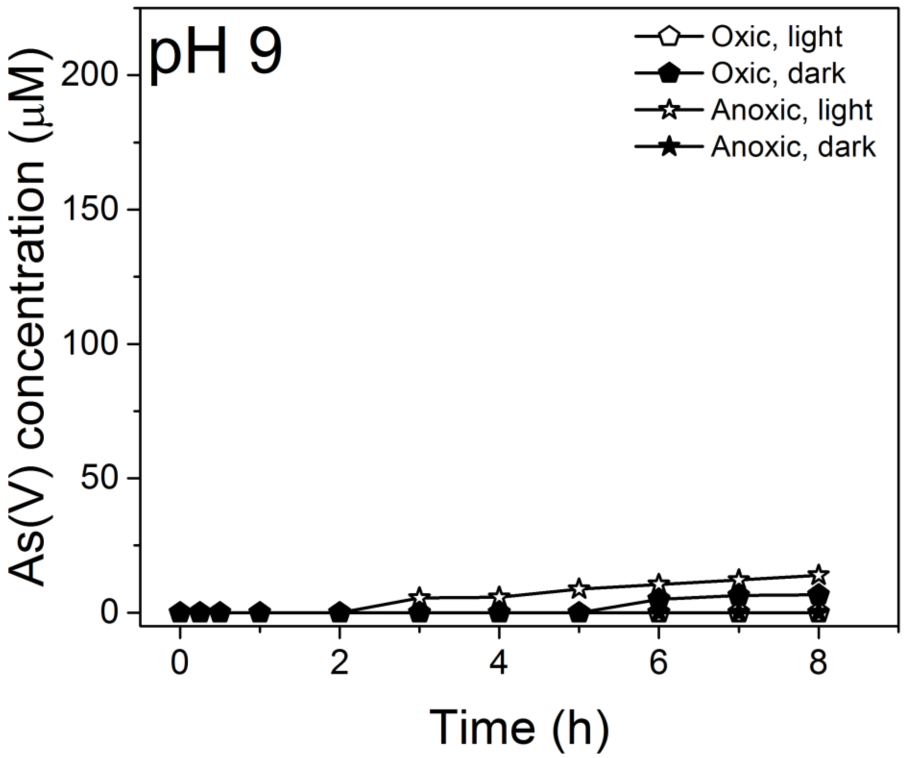

Figure S5: Concentration of aqueous As(V) formed in solution at pH 9 in the presence of light and absence of light, under oxic and anoxic conditions.

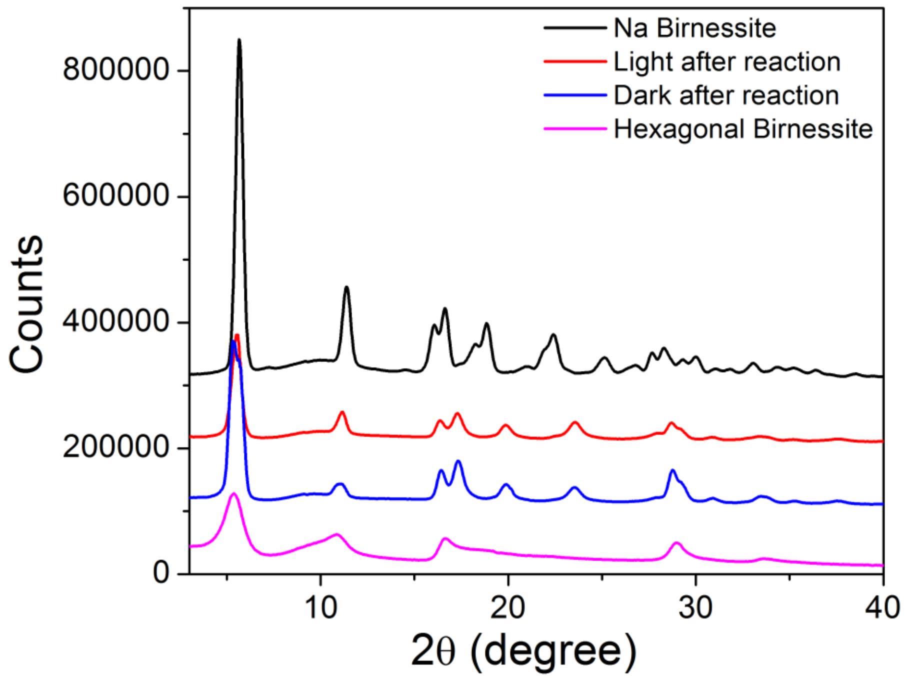

Figure S6: XRD of Na-birnessite starting material, Na-birnessite after 8 hr reaction with light and As(III) at pH 5, Na-birnessite after 8 hr reaction with As(III) in the dark at pH 5, and a poorly crystalline hexagonal birnessite.

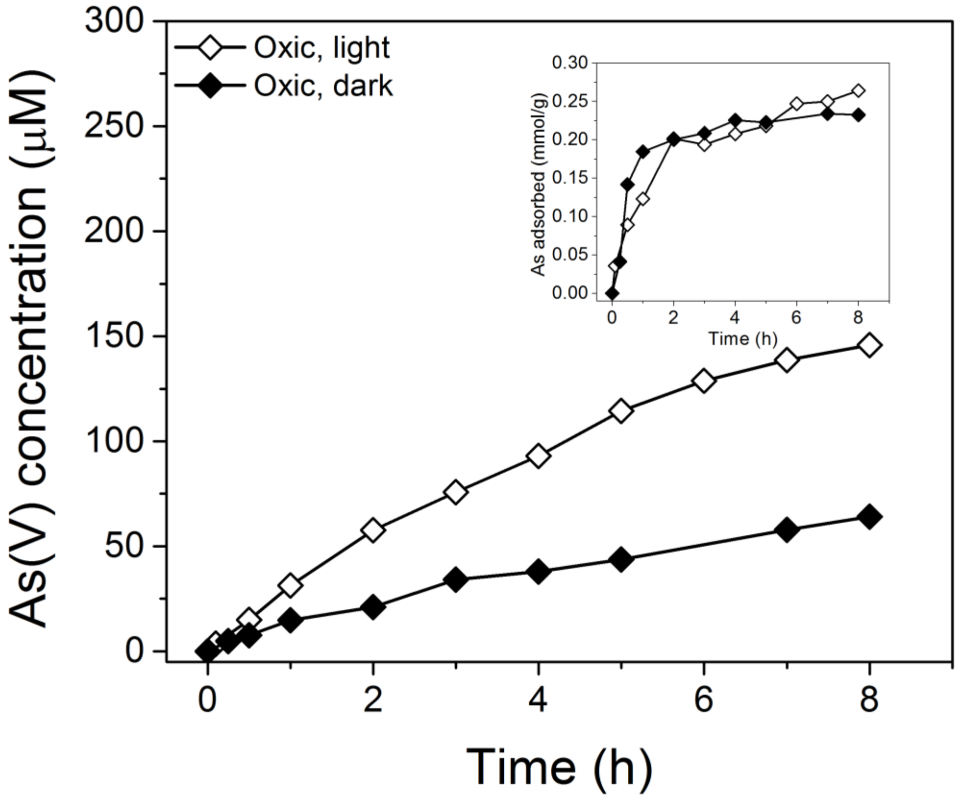

Figure S7: HSA birnessite used for XANES analysis. As(V) production in the presence of birnessite and As(III) under oxic conditions in the presence and absence of light. Inset is the observed As(V) sorption with time. BET surface area of this birnessite sample was 23.10 m^2/g.

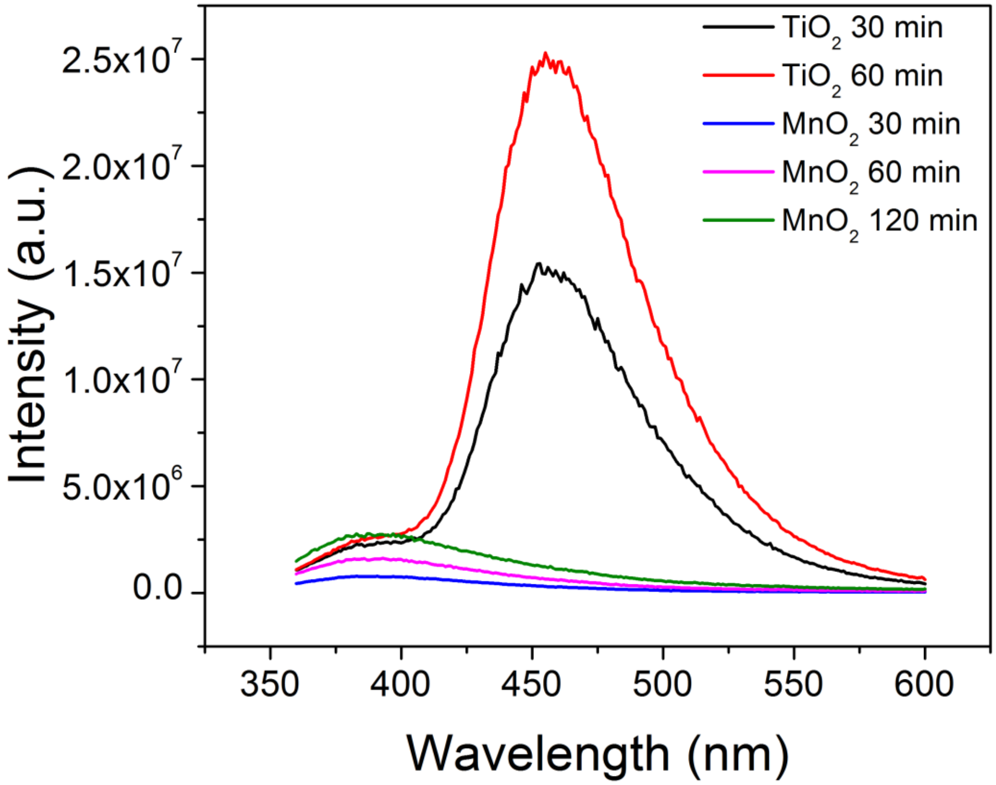

Figure S8: Fluorescence spectra from TiO_2 and MnO_2 suspensions in the presence of coumarin and light. The loading of birnessite was 0.3 mg/L while the TiO_2 loading was 0.15 mg/L. The excitation wavelength used for the experiment was 332nm and the emission wavelength for the coumarin-OH adduct was 460nm. TiO_2 was investigated as a control experiment, since it is well known that TiO_2 generates $^{\bullet}OH$ upon exposure to light [1]. MnO_2 suspensions contained As(III). There was no evidence of $^{\bullet}OH$ in this instance. We also did not detect $^{\bullet}OH$ in an irradiated birnessite suspension in the absence of As(III) .

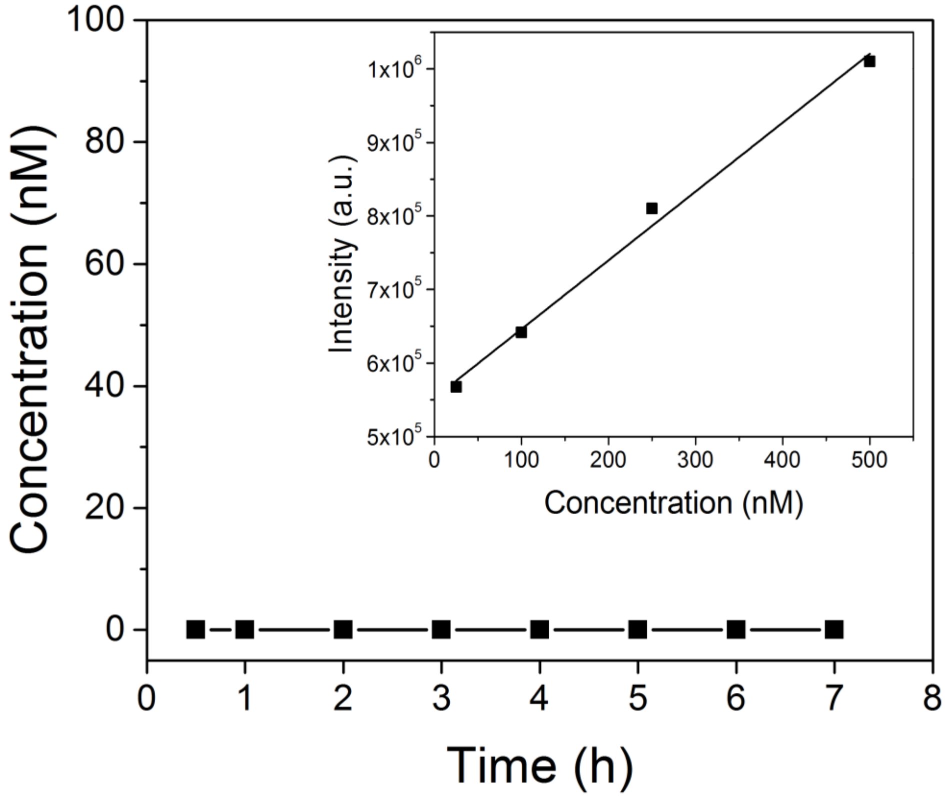

Figure S9: H_2O_2 concentration versus time for a As(III) - birnessite suspension that was exposed to light. The APF-HRP method was used to detect H_2O_2. No H_2O_2 was detected after a suspension of birnessite (no As(III)) was irradiated in the absence or presence of dissolved oxygen (not shown). The inset shows a calibration curve where the analytical method was applied to known concentrations of H_2O_2.

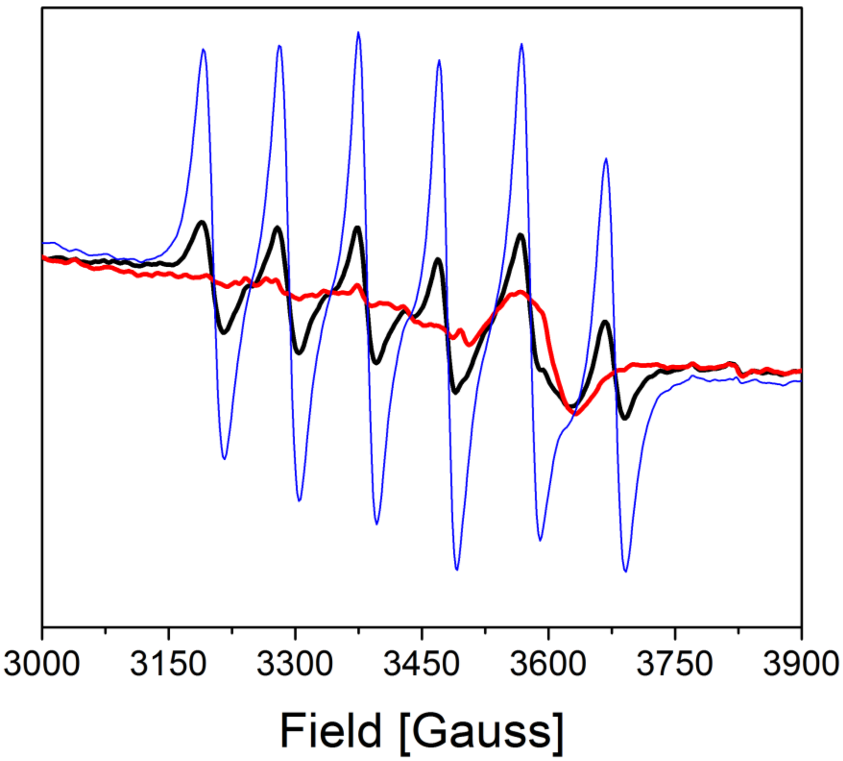

Figure S10: Electron paramagnetic resonance (EPR) spectra of birnessite with As(III) (black) and birnessite without As(III) (red) in the dark. Also shown is a spectrum from a sample containing only Mn^{2+} (blue). The birnessite in the presence of As(III) results in the production of Mn^{2+}.

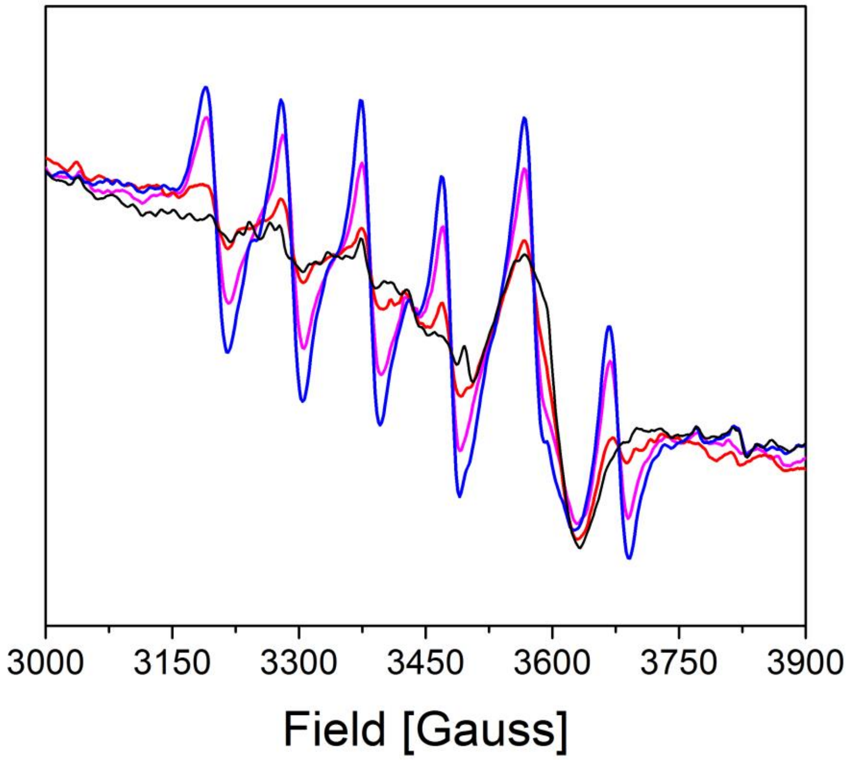

Figure S11: EPR spectra of birnessite at pH 5 under oxic conditions, in the presence of As(III) and DMPO irradiated with light (pink), with As(III) and DMPO in the dark (blue), without As(III) with light (red), without As(III) in the dark (black). All spectra are obtained after 1 h.

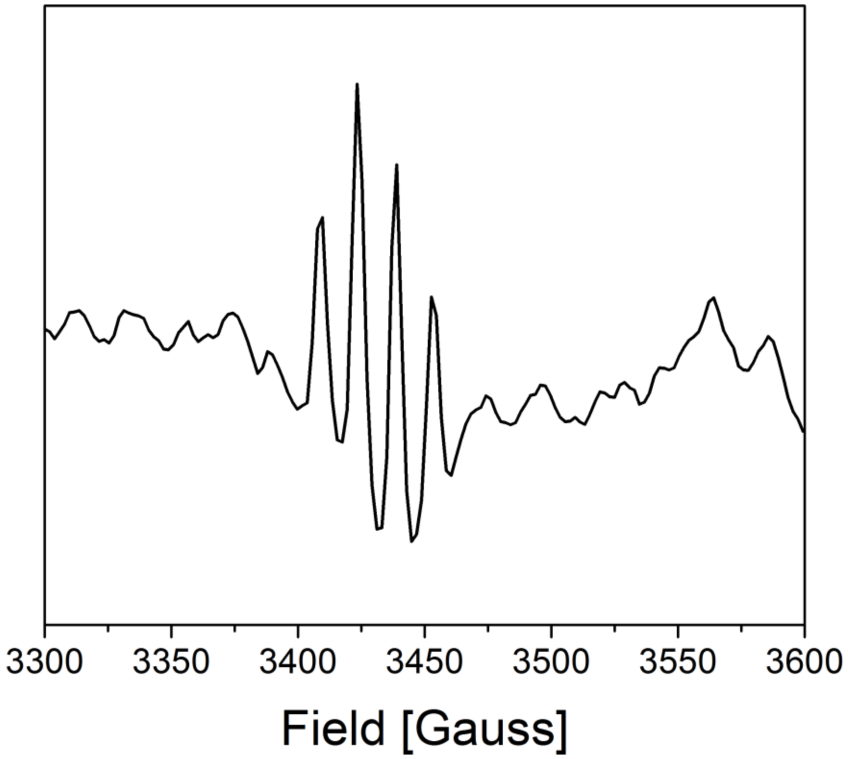

Figure S12: EPR spectrum of control, TiO_2 and DMPO irradiated with light. This spectrum was taken after 10 min irradiation of sample with light, showing evidence of a DMPO-OH spin adduct [2].

References:

[1] A. Wold, Photocatalytic properties of titanium dioxide (TiO_2), Chemistry of Materials, 5 (1993) 280-283.

[2] V. Brezová, S. Gabčová, D. Dvoranová, A. Staško, Reactive oxygen species produced upon photoexcitation of sunscreens containing titanium dioxide (an EPR study), Journal of Photochemistry and Photobiology B: Biology, 79 (2005) 121-134.
